# Lipin1-dependent transcriptional inactivation of SREBPs contributes to selinexor sensitivity in multiple myeloma

**DOI:** 10.1038/s41401-025-01553-3

**Published:** 2025-04-14

**Authors:** Jun-ying Wang, Meng-ping Chen, Jin-xing Jiang, Yi-ke Wan, Xin Li, Yi-wei Zhang, Yi Fang, Hong-hui Huang, Zhao-yu Qin, Jian Hou

**Affiliations:** 1 Department of Hematology, Renji Hospital, Shanghai Jiao Tong University School of Medicine, Shanghai, 200127 China; 2 Key Laboratory of Genetic Engineering and Collaborative Innovation Center for Genetics and Development, School of Life Sciences, Institute of Biomedical Sciences, Human Phenome Institute, Zhongshan Hospital, Fudan University, Shanghai, 200433 China

**Keywords:** multiple myeloma, selinexor, exportin-1, Lipin1, SREBPs, proteomics.

## Abstract

Selective nuclear export inhibitor selinexor (SEL) represents a promising therapeutic strategy for relapsed/refractory multiple myeloma (RRMM). But its mechanisms of action as well as factors that influence therapeutic responses have not been fully characterized yet. In this study we employed catTFRE proteomics technique to profile changes in nuclear abundance of activated transcription factors (TFs)/co-factors (TCs) in myeloma cells following SEL treatment. We found that pharmacological inhibition of exportin-1 (XPO1) by SEL leads to a significant nuclear accumulation of Lipin1 in NCI-H929 cells. Nuclear-localized Lipin1 acted as a transcriptional cofactor that suppressed the transcriptional activity of SREBPs. By performing subcellular localization analysis, molecular docking, co-immunoprecipitation and other assays, we demonstrated that Lipin1 was subjected to XPO1-dependent nuclear export. We demonstrated that SEL downregulated the expression of key lipogenesis-related genes regulated by SREBPs including FASN, SCD, DHCR24 and FDPS, leading to reduced fatty acid and cholesterol synthesis in MM cell lines and primary CD138^+^ cells. Using shRNA-mediated knockdown assays, we elucidated the critical role of Lipin1 in mediating the inhibitory effects of SEL on the SREBPs pathway and its contribution to SEL sensitivity both in vitro and in murine xenograft models. In conclusion, we reveal a novel mechanism by which SEL downregulates cellular lipid biosynthesis, thereby inhibiting the proliferation of myeloma cells. This study highlights the critical role of Lipin1 in the anti-myeloma effects of SEL, suggesting its potential as a biomarker for identifying patients who are most likely to benefit from SEL-based therapies.

## Introduction

Multiple myeloma (MM) is a heterogeneous hematologic malignancy that remains largely incurable [1]. In recent years, selective inhibitors of nuclear export (SINE) have emerged as effective therapeutic agents for relapsed/refractory multiple myeloma (RRMM) [2, 3]. Selinexor (SEL) is the first generation of SINE compounds and has been approved for RRMM in 2019 [4]. Although the combination of SEL with other established drugs can induce remissions in a considerable proportion of patients, some individuals still exhibit poor responses [5–7]. Therefore, from a clinical perspective, it is essential to elucidate the largely undefined mechanisms underlying myeloma inhibition by SEL and to identify biomarkers that can predict treatment responses.

Exportin-1 (XPO1) is a potent transporter that mediates the nuclear export of hundreds of proteins and multiple RNA species [8, 9]. The tumor inhibitory effects of SEL primarily depend on its ability to induce the nuclear retention of various cargo molecules that are transported by XPO1. Previous studies have reported that SINE can retain several crucial tumor suppressor proteins (TSPs) in the nucleus of MM cells, including p53, Rb, p21, and FOXO3a, thereby inducing cell cycle arrest and apoptosis in MM cells [10]. Moreover, SEL downregulates the cytosolic levels of pro-survival proteins, such as Survivin and MCL1 [11]. By inducing the nuclear retention of IκB and TOP2A, SEL can also restore the sensitivity of MM cells to bortezomib and anthracyclines [12, 13]. Although these previous studies have uncovered certain mechanisms behind myeloma inhibition by SEL, the factors that influence sensitivity to SEL remain unclear.

It is widely recognized that the anti-tumor effects of SEL primarily depend on altering the nucleocytoplasmic localization of specific functional molecules transported by XPO1. However, to date, the cargo molecules that might be sequestered in the nucleus of myeloma cells by SEL have not been systematically identified. Using SILAC-based Liquid Chromatography-Tandem Mass Spectrometry (LC-MS/MS) proteomic analysis, Fabio et al. found that SEL had a significant impact on the transcriptional programs in thymic epithelial tumors, with a prominent shift in the abundance of nearly 80 different transcription factors in the nucleus and cytoplasm [14]. Since transcription is one of the most critical biological processes occurring within the nucleus, we speculate that transcription factors (TFs)/co-factors (TCs) may represent a class of cargo molecules contributing to the antitumor effects of SEL [15–17]. However, due to the low abundance of TFs, direct quantitative measurement of these factors on a proteome scale poses a significant challenge. Recently, Ding et al. developed the concatenated tandem array of consensus transcription factor response elements (catTFRE) approach, a novel method that enriches TFs/TCs from nuclear extracts and enables the comprehensive identification of endogenous TFs/TCs on a proteome scale [18]. The catTFRE proteomics detection relies on a synthesized DNA sequence containing the catTFRE motif, which serves as a specialized tool for the efficient enrichment of TFs/TCs from nuclear extracts. By integrating this enrichment strategy with advanced mass spectrometry analysis, the approach facilitates both qualitative and quantitative profiling of TFs/TCs across diverse biological samples.

In this study, we employed catTFRE proteomics techniques to systematically profile changes in the nuclear abundance of activated TFs and TCs in myeloma cells following SEL treatment. Our results revealed a significant nuclear enrichment of the TC molecule Lipin1 post-SEL treatment. Notably, this effect was accompanied by a marked downregulation of the transcriptional activity of sterol regulatory element binding proteins (SREBPs). Lipin1 functions as both a metabolic enzyme and a TC for key regulators of lipid metabolism [19]. SREBPs are a family of TFs that play a crucial role in maintaining lipid homeostasis by regulating the expression of multiple enzymes involved in the synthesis of endogenous cholesterol, fatty acids, triacylglycerols, and phospholipids [20]. Notably, nuclear-localized Lipin1 has been shown to suppress lipogenic programming by repressing the transcriptional activity of SREBPs [21]. Nevertheless, the mechanistic relationship between XPO1-mediated nuclear export and the Lipin1-SREBPs regulatory axis remains to be defined. In this study, using in vitro and in vivo models, we demonstrated that Lipin1 is a cargo molecule of XPO1, which can be retained in the nucleus following XPO1 inhibition treatments. SEL impairs SREBPs-dependent lipogenesis in MM cells in a Lipin1-dependent manner. Furthermore, pharmacological targeting of lipid biosynthesis constitutes a key mechanism underlying the anti-myeloma efficacy of SEL.

## Materials and methods

### Antibodies and reagents

The following antibodies were obtained from Cell Signaling Technology (Danvers, MA, USA): Lipin1 (14906), CRM1 (46249), Lamin B1 (13435), and DYKDDDDK Tag (14793). Antibodies sourced from Proteintech (Wuhan, China) include SREBP1 (14088-1-AP), SREBP2 (28212-1-AP), FASN (10624-2-AP), SCD (28678-1-AP), FDPS (16129-1-AP), DHCR24 (10471-1-AP), α-tubulin (66031-1-Ig), β-actin (66009-1-Ig), Ki67 (27309-1-AP), cleaved caspase-3 (25128-1-AP). Other reagents include Alexa Fluor^®^ 488-conjugated Goat Anti-Rabbit IgG (H&L) (ab150077, Abcam, Cambridge, UK), SEL (S725, Selleck Chemicals, TX, USA), and Recombinant Human Ran Protein (Q69L) (10113, NewEast Biosciences, Wuhan, China).

### Primary MM samples collection and cell culture

Bone marrow samples were collected from participants diagnosed with MM after obtaining written informed consent. All procedures were conducted in accordance with the protocol approved by the Ethics Committee of Renji Hospital, Shanghai Jiao Tong University School of Medicine. Bone marrow mononuclear cells were isolated by density gradient centrifugation using Ficoll-Paque (17544202, Cytiva, MA, USA). CD138^+^ primary myeloma cells were isolated using CD138 MicroBeads (130-051-301, Miltenyi Biotec, Bergisch Gladbach, Germany). The characteristics of the participants are presented in Supplementary Table S1. Human myeloma cell lines and primary myeloma cells were cultured in RPMI-1640 medium (SH30096.02, HyClone Laboratories, UT, USA) containing 10% fetal bovine serum (SFBS-NZ, Bovogen Biologicals, VIC, Australia). Cells were maintained at 37 °C with 5% CO_2_ in a humidified atmosphere.

### Nuclear extractions (NEs) preparation and catTFRE pull-down assay

NCI-H929 cells were subjected to DMSO or 500 nM SEL for 12 h in biological duplicates. After 12 h, cells were collected and washed three times with cold PBS (SH30256.01, Cytiva, MA, USA). Nuclear proteins were extracted using NE-PER^™^ Nuclear and Cytoplasmic Extraction Reagents (78833, Thermo Fisher Scientific, MA, USA) according to the manufacturer’s protocol. Protein concentrations were measured using Bradford method. DNA and biotinylated catTFRE primers were synthesized by Genscript (Nanjing, China). An aliquot of 100 μg NEs was then subjected to catTFRE pull-down assay. In total, 1 pmol of biotinylated DNA was pre-bound to Dynabeads^™^ M-280 streptavidin (11206D, Thermo Fisher Scientific, MA, USA), and then incubated with NEs at 4 °C for 2 h. After incubation, the supernatant was discarded, and the beads were washed with NETN buffer (100 mM NaC1, 20 mM Tris-HCl, 0.5 mM EDTA, and 0.5% NP-40) and PBS twice respectively.

### Protein trypsin digestion and LC-MS/MS analysis

The catTFRE pull-down beads were resuspended with 20 μL SDS loading buffer and boiled for 5 min at 95 °C. The samples were then loaded onto 10 cm 10% sodium dodecyl sulfate-polyacrylamide gel electrophoresis (SDS-PAGE) gels and run for one-third of the gel length. The gel was stained with Coomassie Brilliant Blue and then destained in 5% ethanol and 10% acetic acid solution. Six bands were excised according to the molecular weight ranges and then subjected to in-gel trypsin digestion. 0.1% formic acid was added to stop digestion and 50% acetonitrile was used to extract peptides. Peptide solution was dried in a vacuum concentrator (Thermo Fisher Scientific, MA, USA) and then analyzed by the Q Exactive^™^ HF-X Hybrid mass spectrometer (Thermo Fisher Scientific, MA, USA).

### Bioinformatics and statistical analysis for MS data

The raw MS files were searched against NCBI RefSeq Protein Database. Firmiana one-stop data analysis cloud platform (https://phenomics.fudan.edu.cn/firmiana/login/) was used for qualitative and quantitative analysis. The digestion enzyme was set as trypsin. The peptide mass tolerance was set to 20 ppm, and fragment mass tolerance was set to 0.5 Da. Up to two missed cleavages were allowed for protease digestion. Carbamidomethylation of cysteine was set as a fixed modification, while N-terminal protein acetylation and methionine oxidation were set as variable modifications. For quantitative analysis, the intensity-based absolute quantification (iBAQ) algorithm was applied, which normalizes protein abundance by dividing summed peptide intensities by the number of theoretically observable peptides. Protein abundance was quantified as the fraction of total (FOT), calculated by normalizing iBAQ values against the total proteome intensity [22].

Hierarchical clustering was performed using the pheatmap package (version 1.0.8) in R. Functional enrichment analysis of differentially expressed genes was conducted using the clusterProfiler package (version 4.6.0) in R. Pathway enrichment was considered significant if associated with *P* < 0.05. The networks among TFs and TCs were obtained from the STRING database, and visualization was performed using Cytoscape (version 3.9.0).

### Immunofluorescence assay

MM cells were treated with 500 nM SEL or DMSO for 12 h, then cells were collected and washed twice with cold PBS. Cytospin centrifuge (Thermo Fisher Scientific, MA, USA) was used to prepare cellular smears. Cells were fixed in 4% paraformaldehyde (BL539A, Biosharp, Beijing, China) for 30 min and permeabilized with 0.1% Triton X-100 (P0096, Beyotime, Shanghai, China) for 15 min. After blocking in 3% bovine serum albumin (ST023, Beyotime, Shanghai, China) for 1 h, cells were incubated with the primary Lipin1 antibody at 4 °C overnight, followed by incubation with the secondary antibody for 1 h at room temperature. Nuclei were stained with DAPI and the staining was visualized using immunofluorescence microscopy (Olympus, Tokyo, Japan).

### Subcellular fractionation and Western blotting

Cells were treated with 500 nM SEL or DMSO for 12 h, nuclear and cytoplasmic proteins were extracted as described above. Western blotting was performed as previously described [23]. Total cell protein was extracted using RIPA lysis buffer (P0013B, Beyotime, Shanghai, China) supplemented with protease and phosphatase inhibitors (P1005, Beyotime, Shanghai, China). Proteins were separated by SDS-PAGE and transferred to polyvinylidene fluoride membrane (Millipore, Burlington, MA, USA). Membranes were blocked with 5% BSA for 1 h and then incubated with primary antibodies at 4 °C overnight. On the next day, membranes were incubated with a secondary antibody for 1 h and detected using enhanced chemiluminescence (Millipore, Burlington, MA, USA).

### NES domain analysis and molecular docking

The amino acid sequence of Lipin1 was retrieved from the NCBI database and submitted for NES domain prediction using the online computational tool LocNES (http://prodata.swmed.edu/LocNES/LocNES.php). Structure of XPO1-RanGTP (ID: 3NC1) was downloaded from the Protein Data Bank (PDB) database and the Lipin1 protein structure (Uniport ID: Q14693) was obtained from the Uniport database. Protein was preprocessed using PyMOL (version 2.4.0), including removal of water molecules and redundant ligands, as well as the addition of hydrogen atoms. The HDOCK server (http://hdock.phys.hust.edu.cn/) was utilized for molecular docking of protein-protein interactions [24]. The docking score, confidence score, and root-mean-square deviation (RMSD) were employed as the evaluation criteria for the docking results, with the top 10 docking positions being output. The model with the highest score was selected as the optimal docking model. Finally, the docking results were visualized using PyMOL.

### Co-immunoprecipitation (Co-IP) assay

Co-IP was conducted using IP/Co-IP Kit (abs955, Absin, Shanghai, China) in accordance with the manufacturer’s instructions. Briefly, cell lysates from 2 × 10^7^ cells were prepared with lysis buffer supplemented with protease inhibitors (Beyotime, Shanghai, China). Then, the lysates were incubated with primary antibodies against bait proteins overnight at 4 °C. On the next day, 5 μL Protein A/G agarose beads were added to the sample and incubated at 4 °C on a rotator for another 3 h. Then, the beads were washed three times with 1× wash buffer, proteins were eluted using 2× loading buffer (Beyotime, Shanghai, China) at 95 °C for 5 min. Then proteins in the immunoprecipitates were analyzed by Western blotting.

### Real-time quantitative PCR (RT-qPCR)

RNA was extracted using RNeasy Mini Kit (74106, QIAGEN, Hilden, Germany), followed by reverse transcription. RT-qPCR was performed with ChamQ Universal SYBR qPCR Master Mix (Q711, Vazyme Biotech Co., Ltd, Nanjing, China) in accordance with the manufacturer’s instructions. qPCR standard protocol was 95 °C for 5 min, followed by 45 cycles of 95 °C for 10 s and 60 °C for 30 s. Expression fold change (FC) was calculated using 2^−ΔΔCt^. The primer sequences used in this study are listed in Supplementary Table S2.

### Intracellular fatty acid and cholesterol detection

Approximately 5 × 10^6^ cells were collected and washed twice with cold PBS. Then, cells were resuspended in 100 μL PBS and cellular homogenate was prepared utilizing ultrasonic cell disruptor. Fatty acid and cholesterol content in cell homogenate was detected with Nonesterified Free Fatty Acids Assay Kit (A042-2-1, Jiancheng Bioengineering Institute, Nanjing, China) and Total Cholesterol Assay Kit (A111-2-1, Jiancheng Bioengineering Institute, Nanjing, China) according to the instruction manuals respectively.

### Lentivirus vectors and cell infection

shRNA-encoding plasmids were obtained from the Thermo Scientific Open Biosystems GIPZ Lentiviral shRNA Library. Flag-tagged LPIN1 and myc-tagged SREBF1/2 expression plasmids were obtained from Genechem (Shanghai, China) and cloned into the pBOSLV3-CMV-MCS-EF1a-puro vector. Lentiviral particles were constructed by co-transfecting HEK293T cells with the transfer plasmid and packaging plasmids using jetPRIME^®^ transfection reagent (101000046, Polyplus-transfection SA, Illkirch, France) according to the manufacturer’s instructions. After 48 h, virus containing supernatants were collected and filtered through 0.45 μm cellulose acetate filters. MM cells were plated in 6-well plates at 60%–70% confluence and infected for 12 h in the presence of 8 μg/mL polybrene. After infection, the cells were replaced with fresh media. Knockdown efficiency was verified by RT-qPCR and Western blotting. LPIN1, XPO1, SREBF1 and SREBF2 shRNA sense sequences were listed in Supplementary Table S3.

### Cell viability assay

Cells were seeded in 96-well plates with respective treatments and incubated at 37 °C. 10 μL Cell Counting Kit-8 (CCK-8) solution (CK04, Dojindo Laboratories, Kumamoto, Japan) was added to each well. After incubation for 3 h, absorbance at 450 nm was measured by a microplate reader (Thermo Fisher Scientific, MA, USA). Data represent the mean ± SD of biological triplicates.

### Cell cycle profiling and apoptosis assays

MM cells were treated with DMSO or SEL for the indicated times. For cell cycle analysis, cells were stained with PI/RNase Staining Buffer (550825, BD Pharmingen, CA, USA), and DNA content was measured using flow cytometry (CytoFLEX, Beckman Coulter, CA, USA). The proportion of cells in G_0_/G_1_, S, and G_2_/M phases was determined using ModFit LT software (Verity Software House, ME, USA). Apoptosis was assessed using the PE Annexin V Apoptosis Detection Kit (559763, BD Pharmingen, San Diego, CA, USA) and analyzed by flow cytometry.

### MM xenograft mouse model

Female NOD-SCID mice (aged 6-8 weeks) were obtained from Sibefu Biotechnology Co., Ltd (Beijing, China). Mice were subcutaneously injected in the right flank with 5 × 10^6^ NCI-H929 cells stably transduced with LPIN1-targeting shRNA (shLPIN1) or scrambled control shRNA (shNC). When the established tumors became palpable, the mice were randomly assigned to receive either PBS (vehicle control) or SEL at a dosage of 10 mg/kg via oral gavage (OG) twice weekly (BIW). Body weight and tumor sizes were monitored every 3 days. Tumor sizes were measured using a caliper, and the volume was calculated using the following formula: 0.5 × width^2^ × length. On day 28, the mice were sacrificed. The tumor tissues were harvested, weighted, photographed, and used for immunohistochemical analyses. The animal experiments in this study were approved by the Animal Ethics Committee of Renji Hospital, Shanghai Jiao Tong University School of Medicine. All animal experimental procedures were conducted in strict accordance with the NIH Guide for the Care and Use of Laboratory Animals.

### Immunohistochemistry analysis

Tumor tissues were fixed with 4% paraformaldehyde and embedded with paraffin. Tumor tissue sections were prepared at a thickness of 4 μm and were placed on glass slides. Slides were incubated with primary antibody overnight at 4 °C. Subsequently, the slides were incubated with the secondary antibody at 37 °C for 45 min. Images were captured using a Leica DMI 6000B microscope (Leica Microsystems GmbH, Wetzlar, Germany).

### Statistical analysis

Statistical analysis was conducted with GraphPad Prism (version 8.0). All data are presented as mean ± SD of three independent experiments of technical triplicates unless specifically stated otherwise. Unpaired two-sided *t*-tests were used to compare two experimental groups, while one-way ANOVA tests were utilized for comparing three or more experimental groups. *P*-values less than 0.05 were considered statistically significant (^*^P < 0.05, ^**^P < 0.01, ^***^P < 0.001, and ^****^P < 0.0001).

## Results

### catTFRE proteomics analysis revealed SEL-induced nuclear accumulation of TC molecule Lipin1 in MM cells

**Fig. 1:**
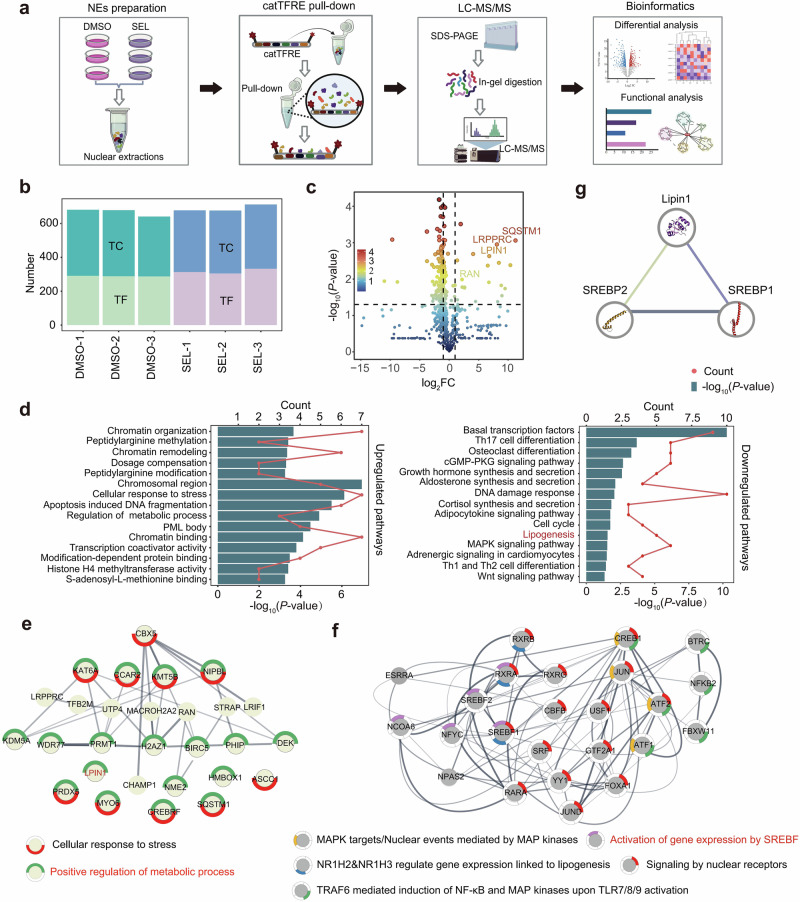
catTFRE proteomics analysis revealed SEL-induced nuclear accumulation of TC molecule Lipin1 in MM cells. **a** Schematic illustration of the experimental procedure for catTFRE proteomics detection. NCI-H929 cells were treated with either DMSO (*n* = 3) or 500 nM SEL (*n* = 3) for 12 h followed by nuclear protein extraction, catTFRE pull-down, mass spectrometry detection and bioinformatics analysis. **b** The bar plot indicated the number of TFs and TCs detected in each sample. **c** The volcano plot illustrates the significantly altered TFs/TCs in SEL-treatment group compared to DMSO-treatment group. **d** Pathway enrichment analysis of upregulated and downregulated TFs/TCs in MM nucleus following SEL treatment. **e**, **f** Molecular network of upregulated (**e**) or downregulated (**f**) TF/TCs, along with the biological processes in which they are involved. **g** Protein-protein interaction analysis revealed that Lipin1 interacts with SREBP1 and SREBP2.

The workflows for the catTFRE proteomics screen are illustrated in Fig. 1a. In total, more than 600 TFs/TCs were identified by mass spectrometry in each sample (Fig. 1b). Components from various TF families, including bHLH, E2F, Homeobox, MYB, ETS, Fork-head, were all detected (Supplementary Fig. S1a). Qualitative analysis showed that 426 TFs and 326 TCs were identified in the control group, with 40 TFs and 20 TCs uniquely detected. In contrast, 410 TFs and 361 TCs were identified in the SEL-treated group, including 24 TFs and 55 TCs that were uniquely identified (Supplementary Fig. S1b). The differential analysis results shown in Supplementary Fig. S1c indicate that 7 TFs and 18 TCs were significantly upregulated in the SEL treatment group. Conversely, the DNA binding activity of 48 TCs and 99 TFs were significantly decreased in the SEL treatment group (|log_2_FC | ≥ 1.2, *P*-value < 0.05). These results indicated that SEL had a deep impact on the transcriptional programs in MM cells, with a prominent shift in the abundance of nearly 170 different TFs/TCs in the nucleus.

SEL directly inhibits the nuclear export of cargo molecules of XPO1. Thus, we speculate that the significantly upregulated TFs/TCs may serve as potential cargo molecules mediating the anti-myeloma effects of SEL. TCs do not have transcriptional activity, but they can regulate the transcriptional activity of TFs [25]. We found that several of the significantly upregulated TFs/TCs detected in our study have been confirmed to be functionally linked to XPO1 (Supplementary Fig. S1d). For example, SQSTM1 (p62) has been reported to relocalize to the nucleus upon XPO1 inhibition by eltanexor, which plays a vital role in p53 activation [26]. LRPPRC can act as an adapter protein to mediate mRNA nuclear export by XPO1 [27]. Ran is indispensable for the transport function of XPO1, as XPO1 binds its cargoes in a Ran-dependent manner [28]. These results suggest that our approach is proficient at identifying the cargo molecules associated with XPO1. Moreover, we also identified several molecules that have not been previously proved to be linked with XPO1, such as LPIN1 (Fig. 1c).

To investigate the relationship between variations in the nuclear abundance of those TFs/TCs and the anti-myeloma effects of SEL, we analyzed the alterations in transcriptional program induced by SEL. Pathway enrichment analysis showed that the upregulated TFs/TCs are predominantly involved in biological processes such as cellular response to stress, chromatin remodeling, regulation of metabolic process, and cell apoptosis. Meanwhile, the downregulated TFs/TCs were associated with biological processes including osteoclast differentiation, DNA damage response, MAPK signaling pathway, and lipogenesis (Fig. 1d). The regulation of DNA damage response and MAPK signaling pathway has been confirmed to be implicated in the anti-tumor effects of SEL [29, 30]. Whereas the effects of SEL on regulating metabolic process are barely inferred. Given that metabolic dysfunction is increasingly recognized as a crucial factor in the pathobiology of MM, we further investigated whether inhibiting lipogenesis contributes to the anti-myeloma effects of SEL. We conducted molecular network analysis to identify hub molecules involved in the alterations of these transcriptional program. Several significantly upregulated TCs, including LPIN1, SQSTM1, PRDX5, and MYO6, are involved in the regulation of metabolic processes (Fig. 1e). Although these molecules do not exhibit direct transcriptional activity, they can modulate the functionality of TFs. Among the significantly downregulated TFs/TCs, SREBPs play a central role in linking the molecule networks of nuclear receptors signaling, MAPK signaling and lipogenesis pathway (Fig. 1f). Previous studies have found that elevated SREBP1/2 expression may lead to abnormal lipid accumulation and bortezomib resistance in myeloma cells, highlighting the potential of targeting SREBPs for MM treatment [31]. Moreover, increased expression of SREBF1 and SREBF2 in MM cells correlated with significantly shorter progression-free survival (PFS) in both the CoMMpass (*n* = 747) and GSE9782 (*n* = 244) databases (Supplementary Fig. S1e). Therefore, we hypothesize that targeting the SREBPs-dependent lipogenesis pathway is essential for the anti-myeloma efficacy of SEL.

Given that the transcriptional activity of TFs is also modulated by TCs, we investigated whether the upregulated TCs induced by SEL exert an inhibitory effect on SREBPs. The protein-protein interaction analysis revealed that Lipin1 interacts with SREBP1/2 (Fig. 1g). Lipin1 functions both as a metabolic enzyme and a TC [19]. Studies by Peterson et al. demonstrated that nuclear-localized Lipin1 impairs SREBPs function by reducing nuclear SREBPs protein abundance as well as SREBP promoter activity [21]. We speculate that the inhibition of XPO1 by SEL leads to the nuclear accumulation of Lipin1, thereby attenuating the transcriptional activity of SREBPs.

### Lipin1 is subjected to XPO1-dependent nuclear export and can be retained in the nucleus by inhibiting XPO1

**Fig. 2:**
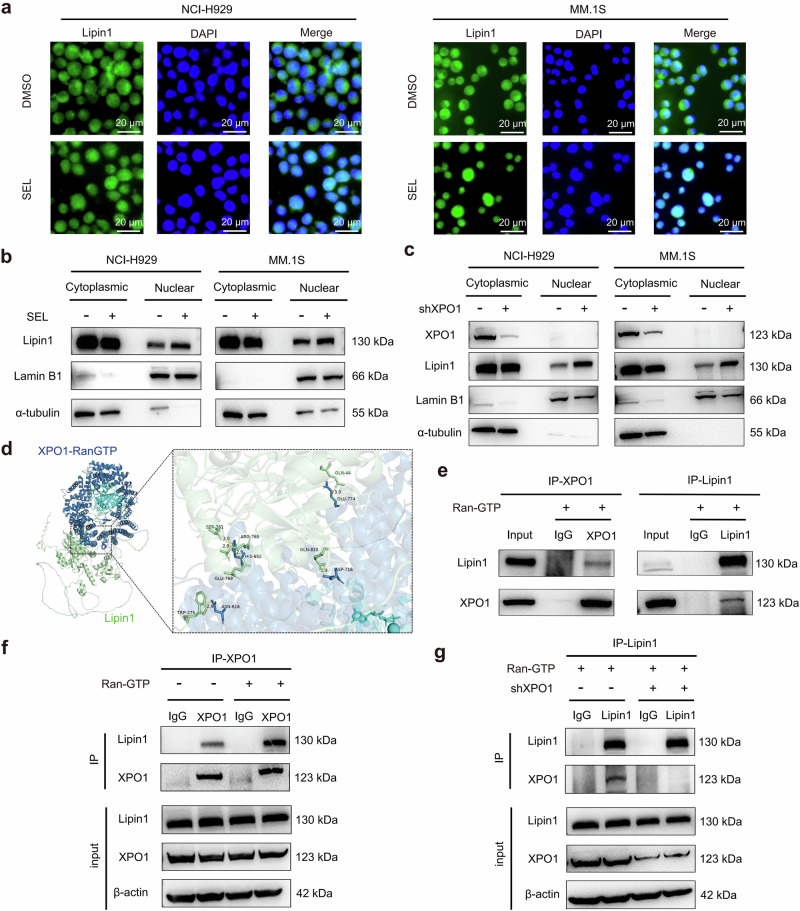
Lipin1 is subjected to XPO1-dependent nuclear export and can be retained in the nucleus by inhibiting XPO1. **a** Immunofluorescence analysis of the subcellular localization of Lipin1 (green) in NCI-H929 and MM.1S following DMSO or 500 nM SEL treatment for 12 h. Cell nuclei were stained with DAPI (blue). Scale bar: 20 μm. **b** Western blot analysis of Lipin1 levels in cytoplasmic and nuclear extractions from MM cell lines treated with DMSO or 500 nM SEL for 12 h. Lamin B1 was used as a loading control for nuclear protein, α-tubulin served as a loading control for cytoplasmic protein. **c** Western blot analysis of Lipin1 levels in cytoplasmic and nuclear extractions from MM cell lines with or without XPO1 knockdown. Lamin B1 was used as a loading control for nuclear protein, α-tubulin served as a loading control for cytoplasmic protein. **d** The molecular docking analysis revealed an interaction between XPO1-RanGTP (upper panel, colored in blue) and Lipin1 (lower panel, colored in green). **e** Co-IP assays were conducted using anti-XPO1 or anti-Lipin1 antibody in cell lysates from NCI-H929 cells, followed by Western blot analysis of the indicated protein levels. **f** Western blot analysis of Lipin1 protein levels in immunoprecipitates derived from NCI-H929 lysates incubated with anti-XPO1 antibody, in the presence or absence of Ran-GTP. **g** Co-IP assays were conducted using anti-Lipin1 antibody in cell lysates from NCI-H929 cells with or without XPO1 knockdown, followed by Western blot analysis of the indicated protein levels.

The nuclear enrichment of Lipin1 following SEL treatment suggests that it may serve as a potential cargo of XPO1. To validate this hypothesis, we conducted immunofluorescence staining and subcellular fractionation assays in NCI-H929 and MM.1S cells, which confirmed SEL-induced nuclear accumulation of Lipin1 (Fig. 2a, b). Furthermore, SEL triggered nuclear translocation of Lipin1 in primary myeloma cells (Supplementary Fig. S2a). To determine whether the nuclear accumulation of Lipin1 directly results from XPO1 inhibition, we knocked down XPO1 expression in MM cells using shRNA (Supplementary Fig. S2b). The results showed that XPO1 knockdown suppressed cell proliferation (Supplementary Fig. S2c) and led to a significant nuclear accumulation of Lipin1 (Fig. 2c), replicating the effects of SEL treatment.

To further elucidate that XPO1 mediates the nuclear export of Lipin1, we employed bioinformatics approaches to identify potential nuclear export signals (NESs) within Lipin1. Using the LocNES tool [32], we identified 20 putative NES candidates in Lipin1, suggesting that it contains recognizable NES sequences (Supplementary Table S4). We also conducted molecular docking to investigate the protein-protein interaction between XPO1-RanGTP and Lipin1 (Fig. 2d). ZDOCK analysis results revealed high affinity scores for the interaction between XPO1-RanGTP and Lipin1 (Supplementary Table S5), suggesting that Lipin1 can effectively bind to XPO1-RanGTP complex. To validate the interaction between XPO1 and Lipin1, we performed immunoblot analysis on anti-XPO1 and anti-Lipin1 immunoprecipitates derived from NCI-H929 cells, with each reaction supplemented with 5 μM Ran-GTP (Ran (Q69L) mutant). The results demonstrated that Lipin1 efficiently co-immunoprecipitated with XPO1 in the presence of Ran-GTP, and vice versa (Fig. 2e). Conversely, the binding of Lipin1 to XPO1 was significantly reduced in the absence of Ran-GTP (Fig. 2f). Additionally, we performed Co-IP assays in NCI-H929 cells with or without XPO1 knockdown. As demonstrated in Fig. 2g, endogenous XPO1 co-immunoprecipitated with Lipin1 in the control cells, while the interaction was completely abolished in XPO1 knockdown cells. Collectively, these findings indicate that Lipin1 is a cargo molecule of XPO1 and can be retained in the nucleus following XPO1 inhibition therapy.

### SEL inhibits the transcriptional activity of SREBPs and downregulates intracellular lipid levels in MM cells

**Fig. 3:**
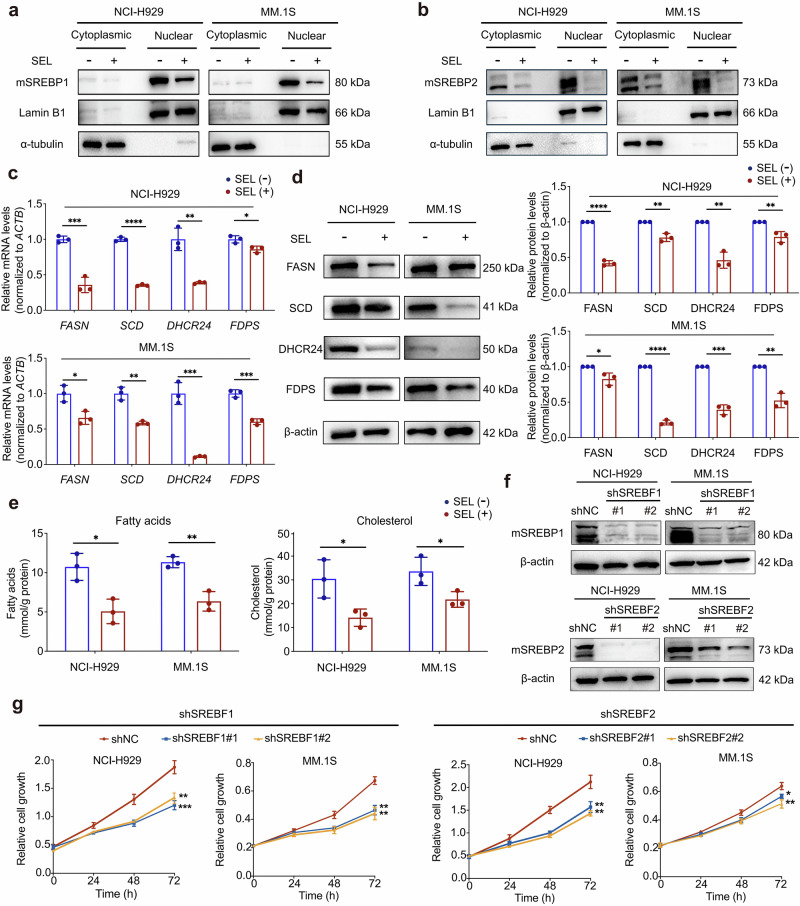
SEL inhibits the expression of SREBPs target genes and downregulates intracellular lipid levels in MM cells. **a**, **b** Western blot analysis of mSREBP1 (**a**) and mSREBP2 (**b**) levels in cytoplasmic and nuclear protein extractions from NCI-H929 and MM.1S cells treated with DMSO or 500 nM SEL for 12 h. Lamin B1 was used as a loading control for nuclear protein, α-tubulin served as a loading control for cytoplasmic protein. **c** mRNA expression analysis of SREBPs downstream targets in MM cell lines treated with 100 nM SEL for 24 h. **d** Western blot analysis of protein levels of SREBPs targets in NCI-H929 and MM.1S cells after treated with 100 nM SEL for 48 h. **e** Colorimetric detection of intracellular fatty acids and cholesterol levels in MM cell lines after treated with 100 nM SEL for 48 h. **f** Western blot analysis of mSREBPs protein levels in MM cells transduced with lentiviral vectors carrying either SREBF1-targeting shRNA (shSREBF1#1, shSREBF1#2), or SREBF2-targeting shRNA (shSREBF2#1, shSREBF2#2), or scrambled control shRNA (shNC). **g** The proliferation of MM cells transduced with shNC or shSREBFs was measured using the CCK-8 assay. Data are shown as mean ± SD; The statistical significance for the above data is indicated as follows: ^*^P < 0.05, ^**^P < 0.01, ^***^P < 0.001, ^****^P < 0.0001.

Our proteomic profiling and bioinformatics analyses suggest that nuclear-localized Lipin1 may function as a suppressor of SREBPs transcriptional activity in MM cells. To validate the regulatory role of SEL in the SREBPs pathway, we conducted further functional assays. We evaluated the nuclear abundance of the mature forms of SREBPs (mSREBPs) following SEL treatment, as these molecules possess transcriptional activity [33]. NCI-H929 and MM.1S cells were treated with DMSO or 500 nM SEL for 12 h, followed by nuclear fraction isolation and Western blotting analysis. The results revealed a significant reduction in nuclear levels of mSREBP1 and mSREBP2 after SEL treatment (Fig. 3a, b). This finding is consistent with previous research suggesting that Lipin1 affects the abundance of mSREBPs in the nucleus [21].

Given the central role of SREBPs in regulating the expression of genes involved in fatty acid and cholesterol biosynthesis, we subsequently detected the mRNA expression of the downstream targets of SREBP1 (FASN, SCD) and SREBP2 (DHCR24, FDPS) [21]. As shown in Fig. 3c, MM cell lines treated with 100 nM SEL for 24 h exhibited a significant downregulation in mRNA expression of SREBP1 and SREBP2 target genes. Furthermore, protein levels of these targets were also markedly decreased after 48 h treatment of 100 nM SEL (Fig. 3d). Additionally, primary myeloma cells also exhibited a significant downregulation of the mRNA expression of SREBPs target genes in response to SEL (Supplementary Fig. S3a). These results indicate that SEL effectively suppresses the transcriptional activity of SREBPs, resulting in a decrease in the expression of critical enzymes implicated in the lipogenesis pathway.

To assess the functional consequences of SREBPs transcriptional inhibition, we quantified the intracellular levels of fatty acids and cholesterol. We found that SEL treatment led to significant reductions in both metabolites (Fig. 3e). Previous studies have shown that proteasome inhibitors can induce abnormal lipid accumulation in MM cells by upregulating the expression of SREBPs. Notably, the combination of lipid-lowering agents with proteasome inhibitors exhibits synergistic cytotoxicity against MM cells [31]. To mechanistically confirm that SREBPs inhibition contributes to the anti-myeloma effects of SEL, we performed shRNA-mediated knockdown of SREBF1 and SREBF2 in MM cells, with knockdown efficiency confirmed by immunoblotting (Fig. 3f). Functional analyses demonstrated that the depletion of SREBP1/2 in MM cells significantly suppressed cell proliferation (Fig. 3g) and promoted cell apoptosis (Supplementary Fig. S3b). Consistent with the proposed mechanism, this depletion also led to a significant reduction in intracellular levels of fatty acids and cholesterol (Supplementary Fig. S3c, d). These findings collectively demonstrate that suppression of SREBPs-dependent lipogenesis represents a critical mechanism underlying the anti-myeloma activity of SEL.

### Lipin1 is essential for the inhibitory effects of SEL on SREBPs-dependent lipogenesis pathway

**Fig. 4:**
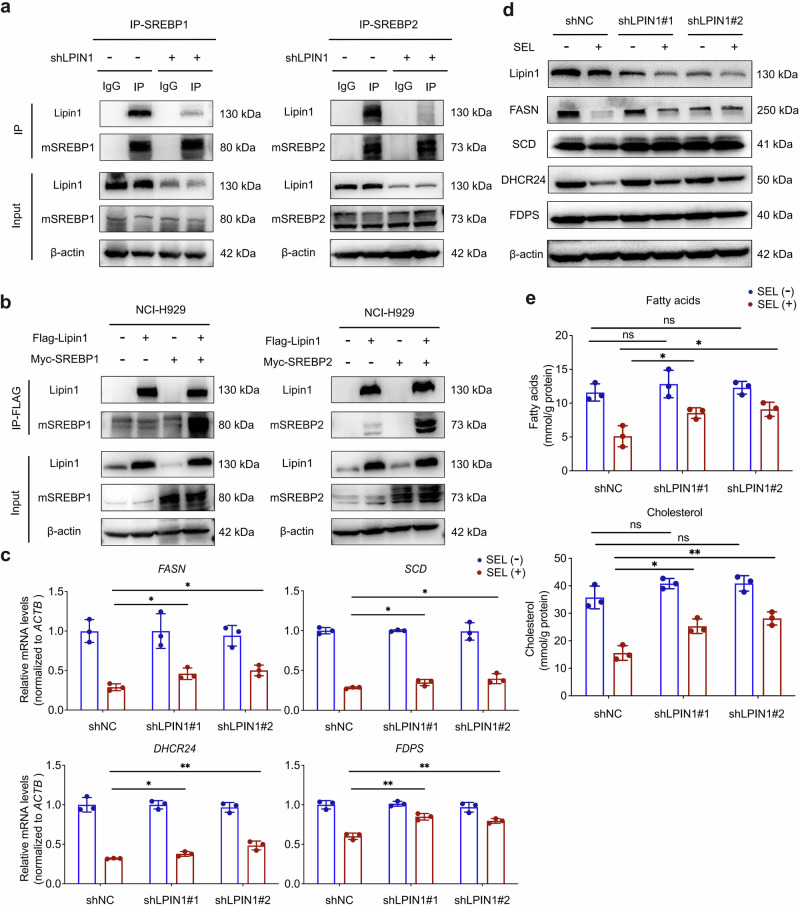
Lipin1 is essential for the inhibitory effects of SEL on SREBPs-dependent lipogenesis pathway. **a** Co-IP assays were conducted in the control and LPIN1-knockdown NCI-H929 cells using anti-SREBP1 or anti-SREBP2 antibodies, followed by Western blot analysis of the indicated proteins. **b** NCI-H929 cells were co-transfected with plasmids encoding Flag-LPIN1 and either myc-SREBF1 or myc-SREBPF2, followed by Co-IP assays and Western blot analysis as indicated. **c** RT-qPCR analysis of mRNA levels of SREBPs targets in NCI-H929 cells transduced with shNC or shLPIN1 after treated with 100 nM SEL for 24 h. **d** Western blot analysis of protein levels of SREBPs targets in NCI-H929 cells transduced with shNC or shLPIN1 after treated with 100 nM SEL for 48 h. **e** Colorimetric detection of fatty acids and cholesterol content in NCI-H929 cells transduced with shNC or shLPIN1 after treated with 100 nM SEL for 48 h. Data are shown as mean ± SD; The statistical significance for the above data is indicated as follows: ^*^P < 0.05, ^**^P < 0.01.

To elucidate the role of Lipin1 in the inhibitory effects of SEL on SREBPs, we knocked down LPIN1 expression in MM cells, the knockdown efficacy was validated at both mRNA and protein levels (Supplementary Fig. S4a, b). We then validated the interaction between Lipin1 and mSREBPs by Co-IP assays. As shown in Fig. 4a, endogenous Lipin1 was co-precipitated with mSREBP1 and mSREBP2 in the control cells, but not in LPIN1 knockdown cells. Furthermore, we conducted rescue assays by reintroducing Flag-tagged Lipin1 and Myc-tagged SREBPs into MM cells. The results confirmed a direct interaction between Lipin1 and mSREBPs (Fig. 4b). Based on the premise that nuclear accumulation of Lipin1 induced by SEL would be abolished in cells lacking Lipin1 expression, we compared the expression levels of the SREBPs targets in MM cells with or without LPIN1 kockdown. This comparative analysis revealed that Lipin1 deletion abolished SEL’s capacity to suppress SREBPs transcriptional programs, as evidenced by a diminished reduction of SREBPs downstream targets in LPIN1 knockdwon cells compared to the control cells under SEL treatment (Fig. 4c, d). Consistently, Lipin1 deletion significantly reversed SEL-induced downregulation of intracellular lipid levels (Fig. 4e). Collectively, these results suggest that the nuclear retention of Lipin1 is essential for the inhibitory effects of SEL on the SREBPs pathway.

### Lipin1 contributes to the sensitivity of myeloma cells to SEL in vitro

**Fig. 5:**
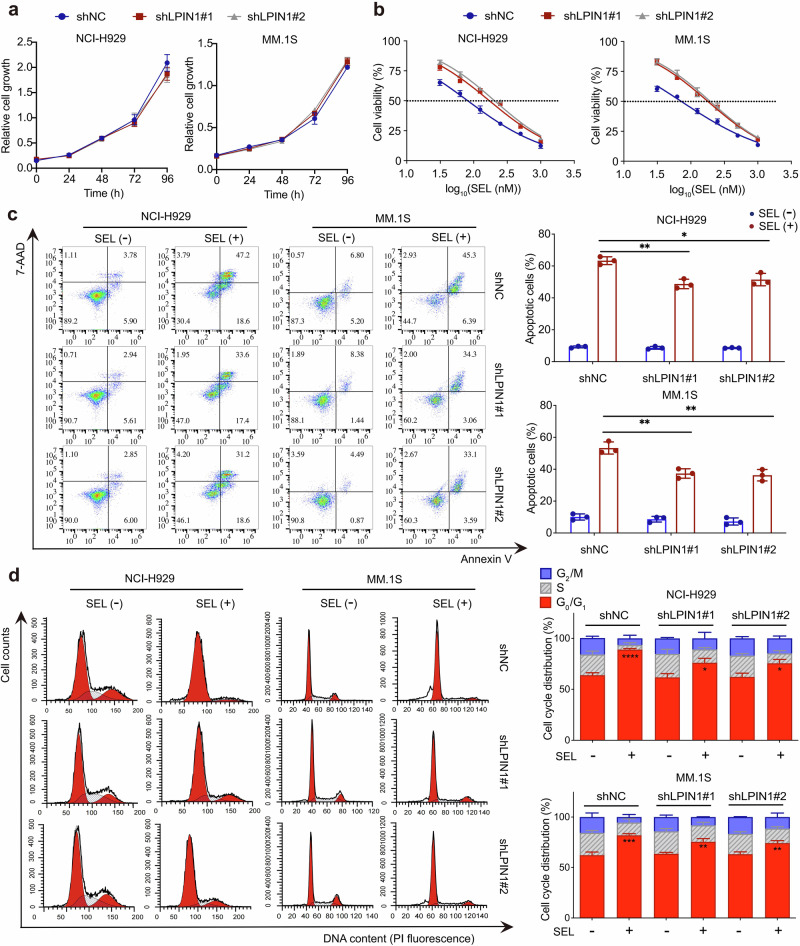
Lipin1 contributes to the sensitivity of myeloma cells to SEL in vitro. **a** MM cells were transduced with lentiviral vectors carrying LPIN1-targeting shRNA (shLPIN1#1, shLPIN1#2) or scrambled control shRNA (shNC). Cell proliferation was assessed using the CCK-8 method. **b** Cell viability of the indicated MM cells treated with increasing doses of SEL was evaluated using the CCK-8 method. **c** Cell apoptosis rates in the control and LPIN1-knockdown MM cells treated with 100 nM SEL for 48 h were analyzed using Annexin V/7-AAD dual staining and flow cytometry. **d** Cell cycle distribution in the control and LPIN1-knockdown MM cells treated with 100 nM SEL for 48 h was analyzed using PI staining and flow cytometry. Data are shown as mean ± SD; The statistical significance for the above data is indicated as follows: ^*^P < 0.05, ^**^P < 0.01, ^***^P < 0.001, ^****^P < 0.0001.

Our results demonstrate that SEL downregulates lipogenesis in myeloma cells by promoting the nuclear accumulation of Lipin1, which inhibits the transcriptional activity of SREBPs. While Lipin1 is often classified as an oncogene in certain cancers, its expression does not exhibit a significant correlation with prognosis in patients with MM (Supplementary Fig. S5a, b). Furthermore, we found that the suppression of Lipin1 had no significant impact on cell proliferation in MM cell lines (Fig. 5a). Subsequently, we investigated whether Lipin1 affects the sensitivity of MM cells to SEL treatment. MM cells with or without LPIN1-knockdown were treated with increasing doses of SEL for 48 h, and the half inhibitory concentration (IC_50_) for each cell line was calculated. The results showed that depletion of Lipin1 resulted in an approximately 2 to 2.5-fold increase in IC_50_ values in NCI-H929 and MM.1S cells (Fig. 5b and Supplementary Table S6). Then, we evaluated apoptosis rates and cell cycle distribution in those cells. As shown in Fig. 5c, suppression of Lipin1 significantly reduced SEL-induced apoptosis. Additionally, SEL-induced G_1_ arrest was also markedly diminished in cells lacking Lipin1 expression (Fig. 5d). In summary, our findings demonstrate that the nuclear accumulation of Lipin1 is the critical determinant of SEL-induced cytotoxicity in myeloma cells.

### Lipin1 enhances the responsiveness of myeloma cells to SEL in vivo

**Fig. 6:**
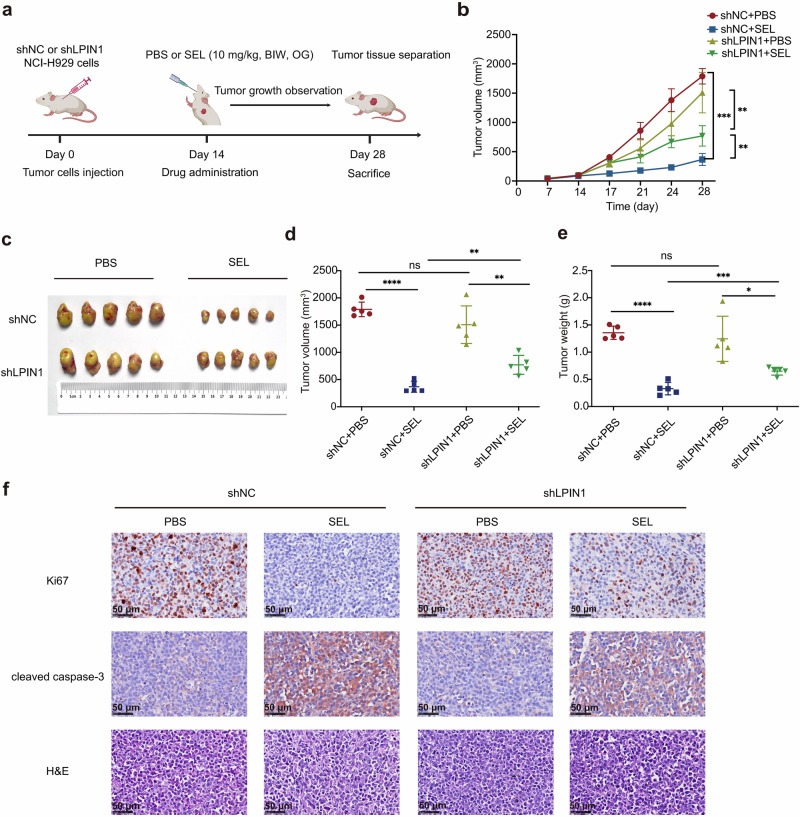
Lipin1 enhances the responsiveness of myeloma cells to SEL in vivo. **a** The timeline and procedures for animal experiments (*n* = 20, *n* = 5/group); BIW: twice weekly, OG: oral gavage. The image was created using BioRender. **b** Tumor growth curves in different groups. **c** Photographic images of terminal tumor xenografts from each group. **d** Quantification of the tumor volume in terminal xenograft from different groups. **e** Quantification of the tumor weights of terminal xenograft in different groups. **f** Immunohistochemical staining of apoptosis marker cleaved caspase-3 and proliferation marker Ki67 expression in xenograft tumor tissues. Scale bar: 50 μm. Data are shown as mean ± SD; The statistical significance for the above data is indicated as follows: ^*^P < 0.05, ^**^P < 0.01, ^***^P < 0.001, ^****^P < 0.0001.

To evaluate the role of Lipin1 in modulating SEL sensitivity in vivo, we established subcutaneous myeloma xenografts in NOD-SCID mice using NCI-H929 cells stably transduced with shNC or shLPIN1. Mice were treated with either PBS or SEL (10 mg/kg, BIW, OG) when the tumors become palpable and were sacrificed approximately two weeks after treatment (Fig. 6a). In PBS-treated mice, tumor growth was unaffected by Lipin1 depletion. Conversely, in SEL-treated mice, xenografts with LPIN1 knockdown exhibited significantly larger volumes and weights, indicating that depletion of Lipin1 compromised the response to SEL treatment (Fig. 6b–e). Immunohistochemical staining demonstrated that Lipin1-deficient xenografts treated with SEL showed a reduction in cleaved caspase-3 expression, along with an increase in Ki67 expression, compared to SEL-treated Lipin1-expressing xenografts (Fig. 6f). These findings conclusively demonstrate that Lipin1 depletion impairs SEL sensitivity in vivo.

## Discussion

Despite significant advances has been made in the treatment of MM, it remains an incurable disease [34]. Pharmacologic inhibition of XPO1 has emerged as a promising therapeutic strategy for the treatment of RRMM [35]. The mechanisms underlying XPO1 inhibition-induced cytotoxic effects in MM cells have not been fully clarified yet. To reach the full potential of XPO1 inhibition therapy in patients with MM, there is an urgent need to establish optimal drug combination regimens as well as reliable predictive biomarkers.

Nowadays, proteomics techniques provide an excellent tool for investigating mechanisms of action of different drugs [36]. To the best of our knowledge, the anti-tumor effects of SEL depend on the reestablishment of tumor suppressor activity through the induction of nuclear entrapment. XPO1 has a wide spectrum of cargoes [37]. Exploring and identifying specific cargo molecules that can be retained in the nucleus of MM cells may help to further elucidate the underlying mechanism of SEL-induced myeloma inhibition. TFs are essential regulators of cellular physiology that function within the nucleus. Restoring the nuclear localization of tumor suppressing TFs, such as p53 and Rb, has been recognized as a vital anti-tumor mechanism of SEL [38, 39]. In the present study, we employed a recently developed proteomic approach, catTFRE, to screen for previously unidentified TFs/TCs targets of SEL in MM cells, aiming to provide valuable insights for enhancing the efficacy of XPO1-targeted therapies.

Through proteomic screening, we found that the TC molecule Lipin1 was significantly enriched in the nucleus after SEL treatment. Concurrently, the transcriptional activity of SREBPs was significantly downregulated in the SEL-treated group. Previous studies have suggested that nuclear-localized Lipin1 inhibits lipogenesis by repressing the transcription activity of SREBPs. With the established link between lipogenesis and carcinogenesis [40–42], we investigated whether the cytotoxic effects of SEL rely on the downregulation of SREBPs-dependent lipogenesis process in MM cells. First, we validated the nuclear accumulation of Lipin1 induced by XPO1 inhibition in MM cell lines and primary MM specimens. Through Co-IP assays and molecular docking, we also confirmed the interaction between Lipin1 and XPO1, demonstrating that Lipin1 is a potential cargo molecule of XPO1. Through measuring the mRNA expression levels of downstream targets transcriptional regulated by SREBPs, we also confirmed that SEL inhibits the transcriptional activity of SREBPs. This inhibitory effect subsequently leads to a reduction in the protein expression of relevant genes, as well as a decrease in the synthesis of fatty acids and cholesterol in MM cells. We then investigated the role of Lipin1 in mediating the inhibitory effects of SEL on SREBPs. By knocking down Lipin1 expression, we disrupted its nuclear accumulation and found that this intervention significantly attenuated the transcriptional inhibition exerted by SEL on SREBPs. In addition, MM cells with Lipin1 depletion exhibited decreased sensitivity to SEL, both in vitro and in xenograft mouse model.

**Fig. 7:**
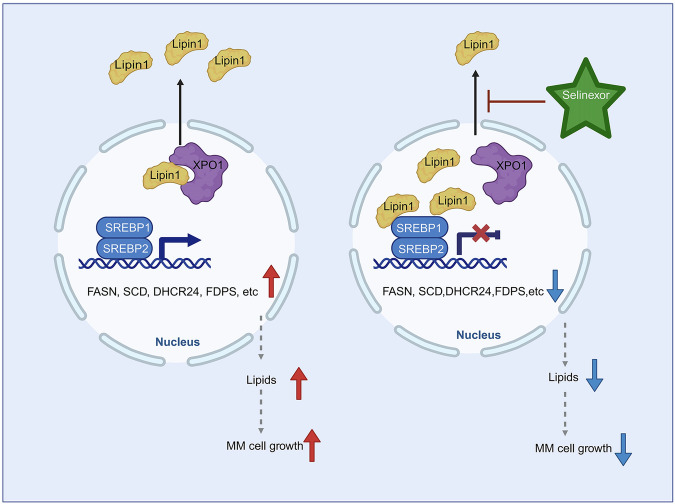
Schematic diagram of the mechanism underlying SEL-induced inhibition of lipogenesis in MM cells. SEL inhibits XPO1, leading to the nuclear retention of Lipin1. In the nucleus, Lipin1 acts as a transcriptional cofactor, suppressing the transcriptional activity of SREBPs. This results in reduced expression of key enzymes involved in the lipogenesis pathway, ultimately decreasing lipogenesis and inhibiting the growth of MM cells. The image was created using BioRender.

Our results indicate that the inhibition of XPO1 significantly impairs SREBPs function in MM cells in a Lipin1-dependent manner. Targeting lipid biosynthesis pathway in MM cells may represent a crucial underlying mechanism of SEL (Fig. 7). We elucidate the anti-tumor mechanism of SEL from a completely new perspective and establish a research foundation for the development of novel targeted combination therapies [43, 44]. Our investigations also identify Lipin1 as a novel predictive biomarker for SEL sensitivity. These results may facilitate the development of more precise and targeted therapeutic interventions, ultimately improving the overall therapeutic outcomes of MM.

## Supplementary information

Supplementary figure

Supplementary table
