# Supplementary material for: Lipin1-dependent transcriptional inactivation of SREBPs contributes to selinexor sensitivity in multiple myeloma
Source: Acta Pharmacol Sin. 2025 Apr 14;46(9):2496–508. doi: 10.1038/s41401-025-01553-3 (PMC12373733; doi:10.1038/s41401-025-01553-3)
Supplement: Supplementary file 1 — Supplementary figure [file 41401_2025_1553_MOESM1_ESM.docx]

**
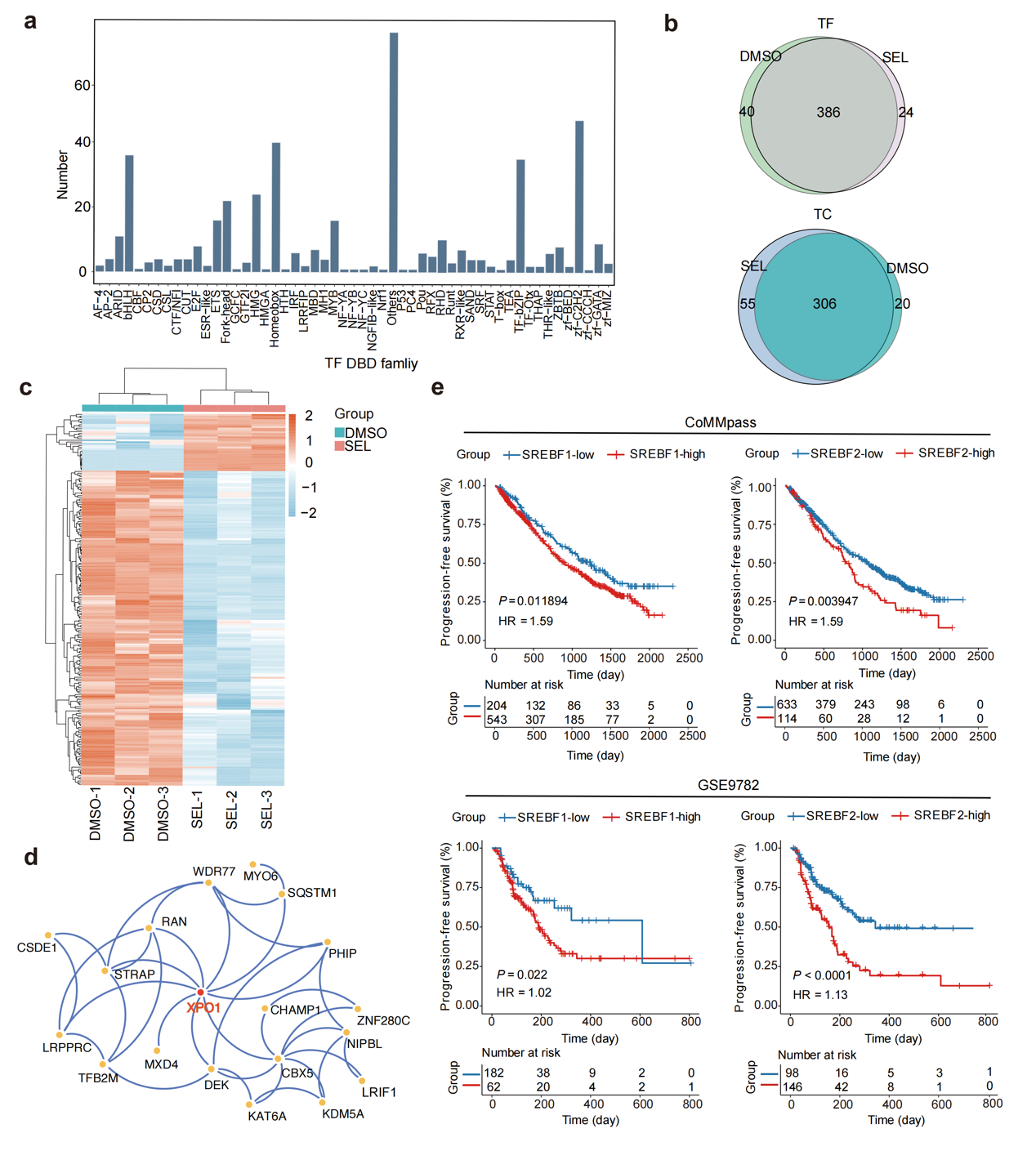
Supplementary Fig. S1 catTRFE proteomics and bioinformatics analysis results.** **a** Numbers of TFs identified in each DNA-binding domains (DBD) family. **b** The Venn diagram illustrates the overlap of detected TFs and TCs between the DSMO-treated and SEL-treated groups. **c** Heatmap shows TFs/TCs with significant differences between the two groups (red: upregulated, blue: downregulated, |log_2_FC | ≥ 1.2, *P <* 0.05). **d** TFs/TCs known to interact with XPO1. **e** Kaplan–Meier analysis showed that MM patients with higher expression levels of SREBF1 and SREBF2 had shorter PFS in CoMMpass (*n =* 747) and GSE9782 (*n* *=* 244) cohorts.


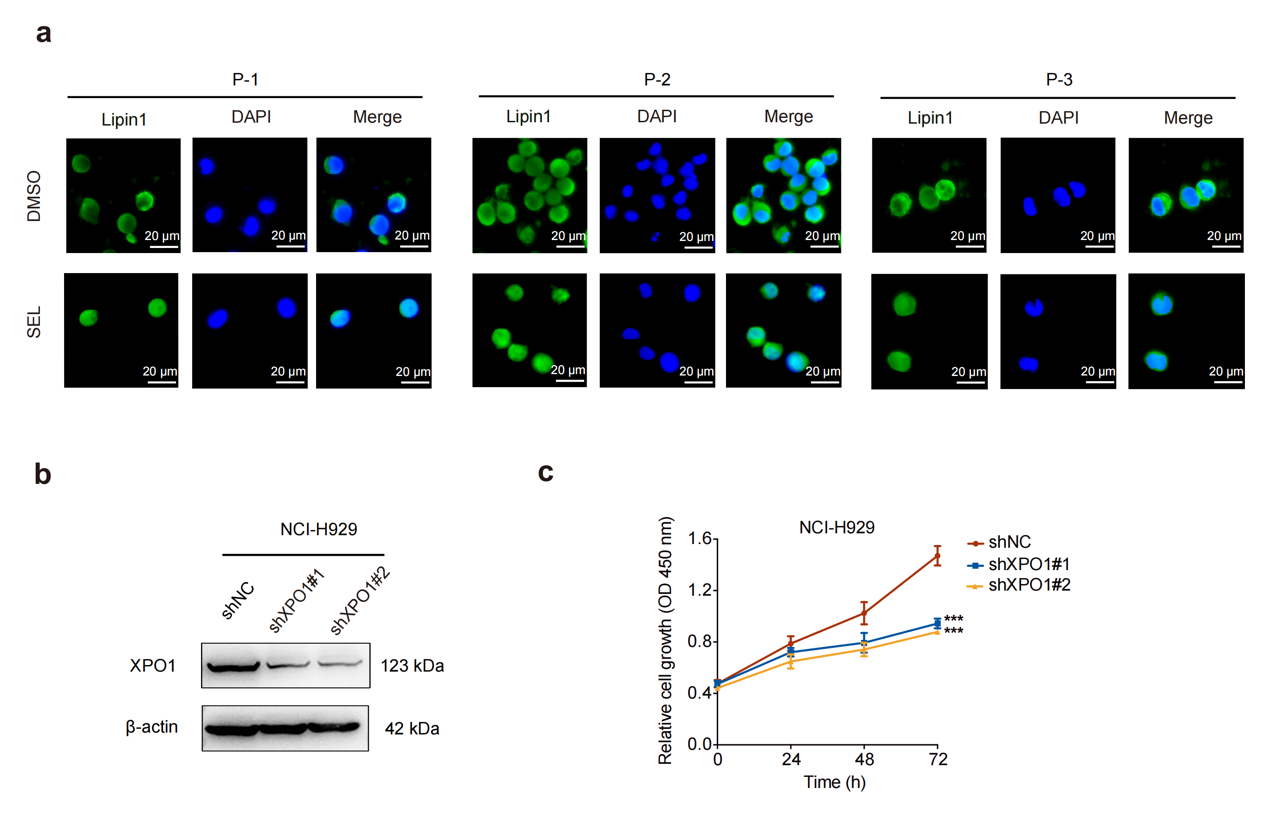
**Supplementary Fig. S2 Inhibition of XPO1 resulted in significant nuclear accumulation of Lipin1. a** Immunofluorescence analysis of the subcellular localization of Lipin1 (green) in primary myeloma cells treated with either DMSO or 500 nM SEL for 12 h. Cell nuclei were stained with DAPI (blue). Scale bar: 20 μm. **b** Western blot analysis of XPO1 levels in NCI-H929 cells transfected with the control scrambled shRNA (shNC) or XPO1-targeting shRNA (shXPO1#1, shXPO1#2). **c** The proliferation of NCI-H929 cells transduced with shNC or shXPO1 was assessed using the CCK-8 method. Data are shown as mean ± SD; The statistical significance for the above data is indicated as follows: ^***^*P <* 0.001.

**
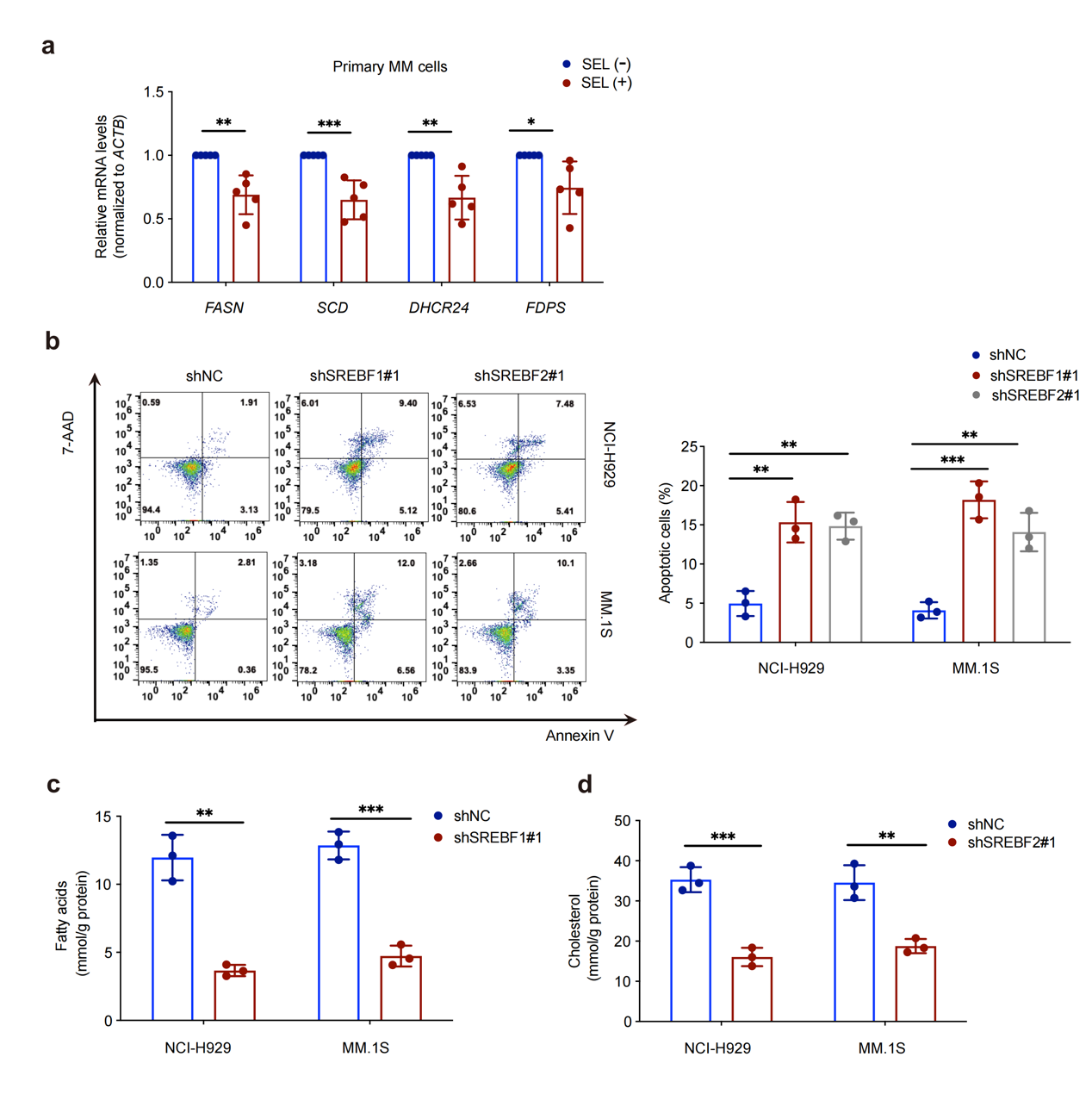
Supplementary Fig. S3 SEL inhibits the expression of SREBPs target genes and downregulates intracellular lipid levels in MM cells.** **a** mRNA expression of SREBPs downstream targets in primary MM cells after treated with DMSO or 100 nM SEL for 24 h (*n* = 5). **b** Cell apoptosis rates in MM cells transfected with lentiviral vectors carrying SREBF1-targeting shRNA (shSREBF1), or SREBF2-targeting shRNA (shSREBF2), or scrambled control shRNA (shNC) were detected using Annexin V/7-AAD dual staining followed by flow cytometry. **c, d** Colorimetric detection of intracellular levels of fatty acids (**c**) and cholesterol (**d**) in indicated MM cells. Data are shown as mean ± SD; The statistical significance for the above data is indicated as follows: ^*^*P <* 0.05, ^**^*P <* 0.01, ^***^*P <* 0.001.

**
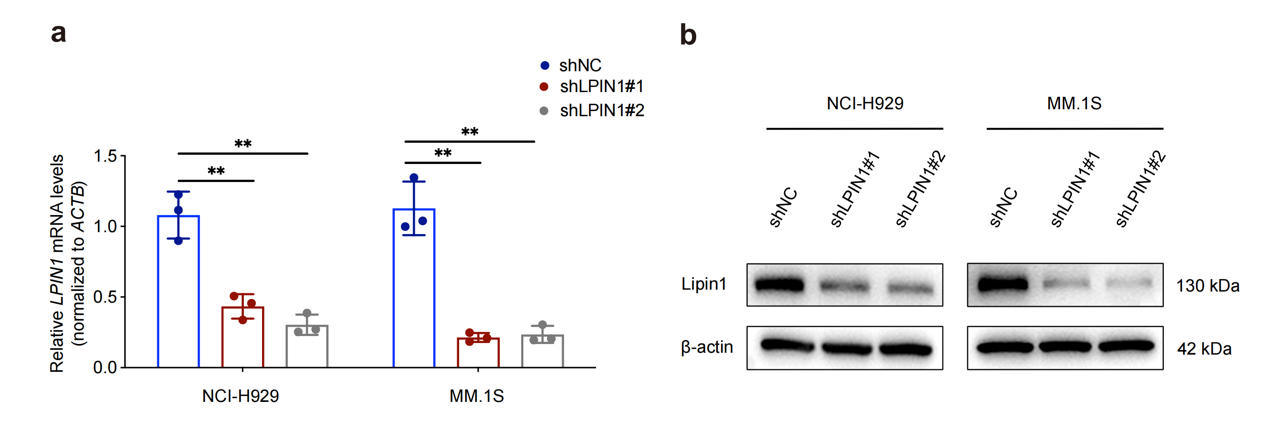
Supplementary Fig. S4 LPIN1 expression levels in MM cells. a, b** N-H929 and MM.1S cells were transduced with lentiviral vectors carrying LPIN1-targeting shRNA (shLPIN1#1, shLPIN1#2) or scrambled control shRNA (shNC). The knockdown efficacy was confirmed at the mRNA level by RT-qPCR (**a**) and at the protein level by Western blotting (**b**). Data are shown as mean ± SD; The statistical significance for the above data is indicated as follows: ^**^*P <* 0.01.


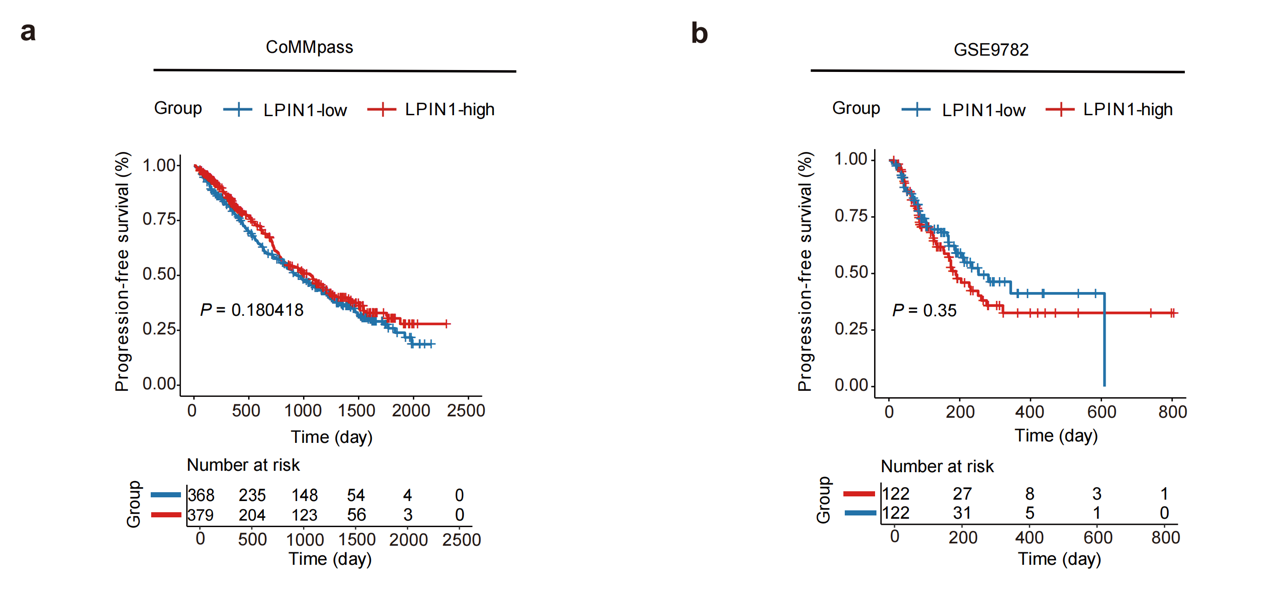
**Supplementary Fig. S5 The correlation of LPIN1 expression and prognosis of MM patients**. **a, b** The expression of LPIN1 shows no significant correlation with PFS in MM patients within the CoMMpass (**a**) and GSE9782 (**b**) cohorts.
