# Supplementary material for: Lipin1-dependent transcriptional inactivation of SREBPs contributes to selinexor sensitivity in multiple myeloma
Source: Acta Pharmacol Sin. 2025 Apr 14;46(9):2496–508. doi: 10.1038/s41401-025-01553-3 (PMC12373733; doi:10.1038/s41401-025-01553-3)
Supplement: Supplementary file 2 — Supplementary table [file 41401_2025_1553_MOESM2_ESM.docx]

**Supplementary Table S1. The characteristics of participants**

| **ID** | **Gender** | **Age** | **M protein** | **Plasma cells (%)** | **Stage** |
| --- | --- | --- | --- | --- | --- |
| P-1 | Female | 67 | λ-Light Chain | 24.5 % | NDMM |
| P-2 | Male | 61 | IgG-κ | 77.5 % | NDMM |
| P-3 | Male | 64 | IgG-λ | 31.5 % | NDMM |
| P-4 | Male | 45 | IgG-κ | 10.5 % | NDMM |
| P-5 | Female | 78 | IgG-λ | 13 % | NDMM |

**Supplementary Table S2. Primers used for RT-qPCR**

| **Gene name** | **Forward sequence (5’-3’)** | **Reverse sequence (5’-3’)** |
| --- | --- | --- |
| *FASN* | GCAAGCTGAAGGACCTGTCT | AATCTGGGTTGATGCCTCCG |
| *SCD* | TCCCGACGTGGCTTTTTCTT | GCCAGGTTTGTAGTACCTCCTC |
| *DHCR24* | GACCTCCATTGGCTGGACTC | GGTCTGAGTTTTCGGACGGA |
| *FDPS* | TGTCCGTTTTGAAGGATGCC | CCTCAGTCAGCACCCTAACG |
| LPIN1 | CCAGCCATGCCTGTCCTAAC | GCCTTGAATTCTGGCAGCTC |
| *ACTB* | GAGGATGAGGTGGAACGTGT | AGAAGTGACGCAGCCCTCTA |

**Supplementary Table S3. shRNA sense sequences**

| **shRNA name** | **Sense sequence (5’-3’)** |
| --- | --- |
| shLPIN1#1 | AGCCTGAAGAGAGATGACA |
| shLPIN1#2 | CCGACCTTCAACACCTAAA |
| shXPO1#1 | GGAAACATTGGTTTATCTT |
| shXPO1#2 | CAGCGAAAGTCTCTGTCAA |
| shSREBF1#1 | GCGGAGAAGCTGCCTATCA |
| shSREBF1#2 | CTGCTTCTGACAGCCATGA |
| shSREBF2#1 | AGGAAGAAGAGAGCTGTGA |
| shSREBF2#2 | AGCTGTGCGCTCTCATTTT |

**Supplementary Table S4. The LocNES prediction results for Lipin1**

| **Position** | **Sequence** | **Score** |
| --- | --- | --- |
| 1-13 | MNYVGQLAGQVFV | 0.018 |
| 4-18 | VGQLAGQVFVTVKEL | 0.020 |
| 22-36 | LNPATLSGCIDIIVI | 0.007 |
| 43-57 | LQCSPFHVRFGKMGV | 0.009 |
| 346-360 | EDLETLGAAAPLLPM | 0.195 |
| 347-361 | DLETLGAAAPLLPMI | 0.209 |
| 350-364 | TLGAAAPLLPMIEEL | 0.105 |
| 385-399 | RDKRSRHLGADGVYL | 0.036 |
| 398-412 | YLDDLTDMDPEVAAL | 0.768 |
| 400-414 | DDLTDMDPEVAALYF | 0.719 |
| 447-461 | VDSGVESTSDGLRDL | 0.145 |
| 452-466 | ESTSDGLRDLPSIAI | 0.173 |
| 515-529 | NWTTAAPLLLAMQAF | 0.207 |
| 624-638 | YKKTLRLTSEQLKTL | 0.243 |
| 626-640 | KTLRLTSEQLKTLKL | 0.467 |
| 715-729 | NGYKFLYCSARAIGM | 0.021 |
| 774-788 | KFKVQCLTDIKNLFF | 0.015 |
| 807-821 | SYKQVGVSLNRIFTV | 0.009 |
| 828-842 | VQEHAKTNISSYVRL | 0.026 |
| 838-852 | SYVRLCEVVDHVFPL | 0.009 |

**Supplementary Table S5. ZDOCK analysis results for XPO1-RanGTP-Lipin1**

| **Docking score** | **Confidence score** | **RMSD** |
| --- | --- | --- |
| -291.23 | 0.9440 | 43.38 |

**Supplementary Table S6. The IC_50_ of SEL in MM cells with LPIN1 knockdown**

| **MM cells** | **NCI-H929**  **shNC** | **NCI-H929**  **shLPIN1#1** | **NCI-H929**  **shLPIN1#2** | **MM.1S**  **shNC** | **MM.1S**  **shLPIN1#1** | **MM.1S**  **shLPIN1#2** |
| --- | --- | --- | --- | --- | --- | --- |
| **IC_50_ (nM)** | 81.33 | 171.4 | 204.8 | 71.79 | 179.3 | 194.5 |
| **95% CI** | 74.92-88.29 | 152.45-192.5 | 187.0-224.2 | 64.95-79.36 | 166.5-193.0 | 173.3-218.3 |
